# Prevalence of Tuberculosis, HIV and Respiratory Symptoms in Two Zambian Communities: Implications for Tuberculosis Control in the Era of HIV

**DOI:** 10.1371/journal.pone.0005602

**Published:** 2009-05-19

**Authors:** Helen Ayles, Albertus Schaap, Amos Nota, Charalambos Sismanidis, Ruth Tembwe, Petra De Haas, Monde Muyoyeta, Nulda Beyers, Peter Godfrey-Faussett for the ZAMSTAR Study Team

**Affiliations:** 1 Department of Infectious and Tropical Diseases, London School of Hygiene & Tropical Medicine, London, United Kingdom; 2 ZAMBART Project, Ridgeway Campus, University of Zambia, Lusaka, Zambia; 3 Department of Epidemiology and Population Health, London School of Hygiene & Tropical Medicine, London, United Kingdom; 4 Chest Diseases Laboratory, Lusaka, Zambia; 5 Desmond Tutu TB Centre, Stellenbosch University, Tygerberg, South Africa; McGill University, Canada

## Abstract

**Background:**

The Stop TB Partnership target for tuberculosis is to have reduced the prevalence of tuberculosis by 50% comparing 2015 to 1990. This target is challenging as few prevalence surveys have been conducted, especially in high burden tuberculosis and HIV countries. Current tuberculosis control strategies in high HIV prevalent settings are therefore based on limited epidemiological evidence and more evidence is needed from community-based surveys to inform improved policy formulation.

**Methods and Findings:**

8044 adults were sampled from 2 sub-districts (wards) in Lusaka province, Zambia. Questionnaires were used to screen for symptoms, respiratory samples were obtained for culture and oral secretions collected for HIV testing. 79 individuals were found to have Mycobacterium tuberculosis in their sputum, giving an adjusted overall prevalence of tuberculosis of 870/100,000 (95% CI 570–1160/100,000). The adjusted overall prevalence of HIV was 28.61% (95% CI 26.04–31.19). HIV- infection was significantly associated with prevalent tuberculosis (Adj OR 2.3, 95% CI 1.42–3.74) and the population attributable fraction of HIV for prevalent tuberculosis was 36%. Symptoms such as prolonged cough (adj OR 12.72, 95% CI 7.05–22.94) and fever (Adj OR 2.04, 95%CI 1.23–3.39), were associated with prevalent tuberculosis, but 8 (10%) individuals with prevalent tuberculosis denied having any symptoms at all and only 34 (43%) would have been classified as a TB suspect by current guidelines.

**Conclusions:**

Undiagnosed tuberculosis is a challenge for tuberculosis control and new approaches are needed if we are to reach international targets. Epidemiological studies can inform screening algorithms for both detection and prevention of active tuberculosis.

## Introduction

Tuberculosis is one of the major causes of morbidity and mortality in poor countries, especially sub-Saharan Africa [1], and is therefore included as one of the diseases covered by the sixth Millennium Development Goal. The target for tuberculosis is to have halted and reversed the spread of tuberculosis by 2015 [2], achievable, according to the Stop TB partnership, only if prevalence of tuberculosis is halved by 2015 compared to 1990 figures. In order to meet this target the Stop TB Partnership has published a new Stop TB Strategy [3]. This strategy acknowledges that DOTS is still the backbone of global tuberculosis control (with targets of finding 70% of new smear positive cases of tuberculosis and curing 85% of them) but that there needs to be increased effort to address the convergence of tuberculosis and HIV, particularly in sub-Saharan Africa where many patients notified with TB are also co-infected with HIV. A WHO TB/HIV policy lays out the activities that national TB and HIV programmes are being encouraged to scale up [4].

Zambia is an example of a country highly affected by HIV with an estimated national HIV prevalence of 17% in adults [5]. Tuberculosis notifications have increased 5- fold in the last 20 years, mainly due to the HIV epidemic. The national TB control programme has expanded DOTS to 100% of government health facilities and has achieved a 76% cure rate and yet has an estimated case detection rate of new smear positive cases of only 53% [6]. Of patients notified with tuberculosis the HIV prevalence is approximately 70%. Zambia has also been in the forefront of rolling out anti-retroviral therapy (ART) to the HIV-infected population. ART became more widely available from 2004 and now more than 120,000 individuals ( representing 50% at need) are receiving it [5].

Neither the target set for monitoring the Millennium Development Goals, nor the case detection rate, key to DOTS, is easy to measure [7]. Few case-finding surveys have been conducted in Africa and most of these were in the pre-HIV era [8], [9], [10], [11], [12], [13]. These early surveys gave invaluable information about the epidemiology of TB, without HIV, and lead to the formulation of the current TB control strategy- DOTS, which relies on passive case-finding by investigating only those individuals presenting to the health services with symptoms of prolonged cough or haemoptysis. However, large population-based surveys have not been conducted during the period since the HIV epidemic has lead to such dramatic increases in the incidence of tuberculosis.

Two prevalence surveys from occupational settings have suggested that HIV may not have such a profound effect on the prevalence of tuberculosis as it does on the incidence [14], [15]. Individuals with HIV have also been shown to be less infectious than those without dual infection [16], [17], but transmission at community level is likely to be directly related to the prevalence of disease in the population. Population-based studies of the risk of transmission of tuberculosis in high HIV prevalent settings have drawn contrasting conclusions with stable risk in East Africa [18] and increased transmission in the gold mines of South Africa [19].

Two recent prevalence studies have been conducted in South Africa. One, from a low HIV prevalence community [20], and another study from a community with high burden of HIV and tuberculosis [21], both in Cape town, indicated that many cases of tuberculosis were not detected by the routine tuberculosis control programme and were only detected by door-to–door screening associated with the surveys. These surveys and a study from a clinical trial site in Tanzania [22] have found that the clinical algorithm used to identify tuberculosis suspects for further investigation, the basis of the DOTS strategy for case-finding, may miss a significant proportion of cases of active tuberculosis.

In this study we present the results of a large community-based tuberculosis and HIV prevalence survey in two communities in Lusaka Province, Zambia. The aim of the study is to determine the prevalence of tuberculosis, HIV and respiratory symptoms and to consider the implications for the new Stop TB Strategy.

## Methods

### Ethics Statement

Ethical approval for the study was obtained from the research ethics committees of the University of Zambia, the London School of Hygiene and Tropical Medicine and Stellenbosch University. Community leaders, district and local health staff and neighbourhood health committees agreed to the study taking place. All individuals involved in the study gave written informed consent.

### Study Sites

The study was conducted in 2 sites (each a political division called a ward) in 2 districts of Lusaka Province of Zambia. The sites were purposively selected to represent rural and urban communities. The rural community has a major road running through it, but otherwise consists of small villages with subsistence farming as the major occupation. The urban community is representative of many such urban settlements in the major towns of Zambia, but not as crowded as the high-density areas in the capital, Lusaka. The majority of the population of this community were employed in small-scale business and informal trading. Each site was served by 1 TB diagnostic centre. The TB notification rates for 2004 were 275/100,000 in the rural site and 438/100,000 in the urban site. The total population according to the 2000 census of the two communities was 17,393 for the rural site and 10,963 for the urban site.

### Sample size calculation

In 2004 WHO estimated the tuberculosis prevalence for Zambia to be 707/100,000[23]. Assuming the prevalence of tuberculosis to be 1000/100,000 among adults we aimed to sample 5000 adults in each site that would give an estimate of prevalence with 95% certainty that the true population prevalence lies somewhere between 810 and 1,190/100,000.

### Sampling strategy

Census maps containing standard enumeration areas were obtained for both of the survey sites. Each standard enumeration area contained between 300 and 2000 adults according to the 2000 census.

As the study was conducted in 2005, five years after the last census, the enumeration data was not considered to be adequate for use in sampling and therefore equal weight was given to each standard enumeration area. All standard enumeration areas within the ward were then listed and assigned a random order. Sampling started with the first randomly assigned enumeration area and all households were visited. All adults≥15 years were enumerated. If adults were not present at the time of enumeration two subsequent appointments were made before the adult was recorded as absent. In the peri-urban area there were 12 enumeration areas and all of these were needed to enumerate 4380 adults. In the rural area there were 22 enumeration areas and 12 were used enumerating 4434 adults.

Following standardised training, research assistants interviewed study participants using a structured paper questionnaire recording socio-economic data (asset register and the number of meals eaten per day), present respiratory symptoms, health seeking behaviour and history of tuberculosis and obtained a spot sputum sample either spontaneously, if the individual was able to, or assisted by simple breathing techniques. Any sample produced, whether it was true sputum or a salivary sample was accepted and sent for culture. An oral fluid sample was also obtained for HIV testing, according to the manufacturer's guidelines (Bionor™ HIV1&2 Test). All individuals were encouraged to attend for counselling and HIV testing using rapid tests on whole blood at the local health centre, where infrastructure and human resource was strengthened to cope with demand.

### Laboratory procedures

All sputum samples were labelled by barcode only and transported in cool boxes to the National TB reference laboratory (Chest Diseases Laboratory, Lusaka) on the same day of collection.

A direct sputum smear was made and stored unstained. This smear was stained using a standard ZN carbol fuschin method and was examined by light microscopy and graded only if the mycobacteria grew on culture (see below).

Samples were decontaminated using the NAOH-NALC decontamination method (BBL™ MycoPrep™ KIT [BD]). Each sample was inoculated onto both a broth (BBL™ MGIT™ 960 [BD]) and solid media (Löwenstein Jensen (LJ) [BD]) and incubated at 37°C either in the automated MGIT960 or a standard incubator for a maximum period of 42 days for MGIT and 56 days for LJ. The remainder of decontaminated sample was stored at −20°C and re-decontaminated if the original culture became contaminated. If mycobacteria grew, confirmed by ZN staining of the culture growth, then the original direct smear was stained and examined. ZN positive cultures were archived in 20% glycerol at −20°C and a DNA sample was obtained for identification by boiling at 96°C for 15 minutes.

Identification of the positive mycobacterial cultures as either *M.tuberculosis* or non-tuberculous mycobacteria (NTM) was done using the Genotype Mycobacteria CM assay (HAIN, Life Science) performed according to manufacturer's instructions.

Oral fluid samples were transported in a cool box to a central laboratory and tested for HIV according to Bionor protocol (Bionor™ HIV1&2 Test) within 3 days of collection.

Laboratory Quality Assurance: All stained sputum smears from individuals with a positive culture were sent to laboratory staff at Stellenbosch University where they were re-read in a blinded manner by 2 individuals. Any discrepancy between the Zambian and South African readers was rechecked by a third panel of readers and a final smear result agreed on.

Positive (*M.tuberculosis* H37RV stock strain) and negative (phosphate buffer alone) culture controls were regularly included to monitor the culture process.

Laboratory cross-contamination was investigated in any case where 2 samples processed on the same day grew *M. tuberculosis*. On these samples spoligotyping was performed [24] and if they had identical spoligopatterns IS*6110* RFLP typing was used to subtype [25].

### Follow up

**Figure 1:**
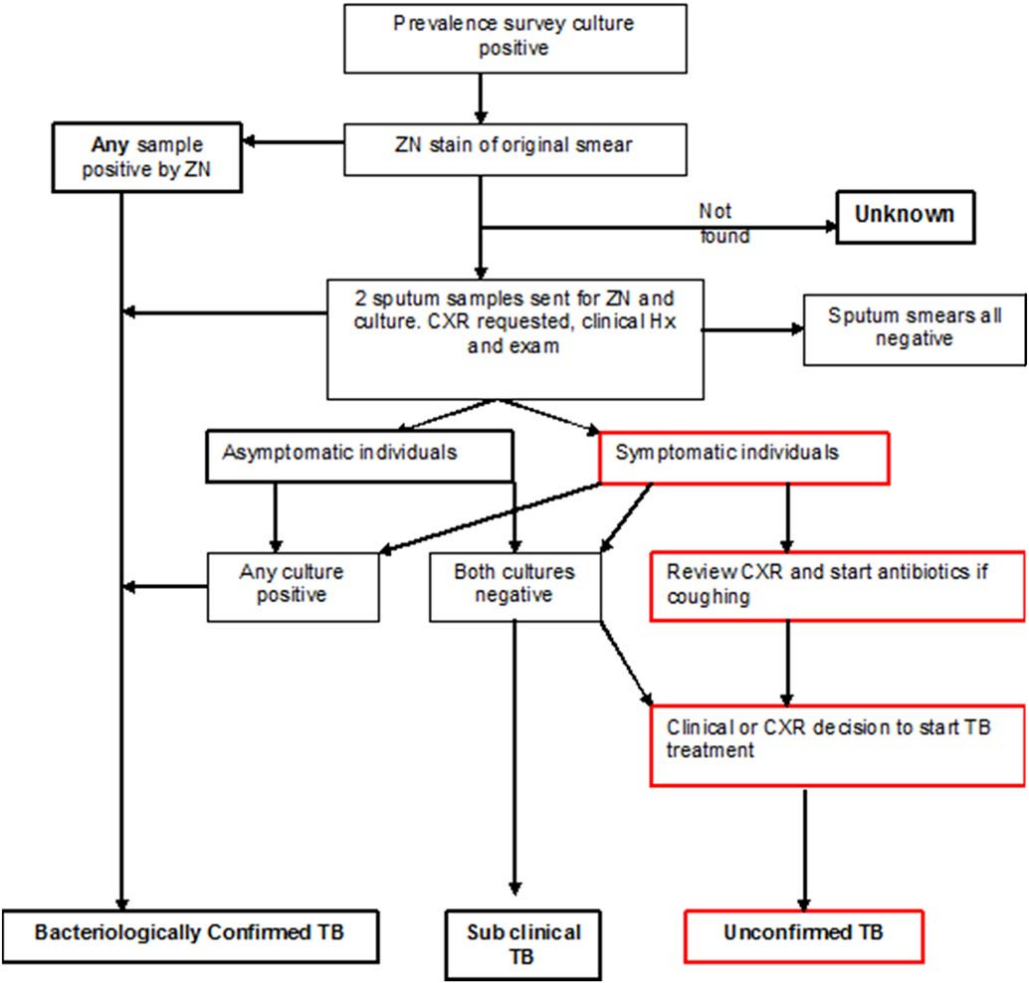
Algorithm for Decision on Clinical TB.

All individuals with a culture positive for Mycobacteria were revisited. Trained clinicians repeated a questionnaire for symptoms and examined the TB suspect. Two further sputum samples (spot and early morning sample) were collected for smear and culture and the TB suspect was transported to the tertiary referral hospital for chest x-ray examination. Any individual suspected or confirmed to have clinical TB according to algorithm (Fig. 1) was started on TB treatment at the local TB diagnostic centre. Counselling and HIV testing were offered to all those found to have TB.

### Data handling

At the first household visits, all household members were enumerated including name, age and sex. Address and GPS coordinates were recorded to ensure possible follow-up at a later stage.

Every participant that consented to the study was assigned a unique study number. Corresponding questionnaire, consent form, sputum sample and oral swab for each individual were labelled and anonymously linked using the bar-coded study number.

Sputum samples were registered electronically at the Zambian National TB-reference Laboratory. The direct smear, decontaminated sputum sample and inoculation tubes of one participant were all coded with the same original barcode number. Positive, negative and contaminated culture results were recorded electronically by scanning the barcode of the culture tube. For positive culture results paper worksheets were printed to capture results of confirmatory tests and smear results. HIV-results were recorded in a register book.

Questionnaire data, laboratory worksheets, HIV results and follow-up questionnaire were dually entered using tailor-made Microsoft Visual Basic 6.0 data entry forms and Microsoft Access 2000 databases. Data entry differences were corrected by referring to the source documents.

### Definitions

#### *M. tuberculosis* prevalence definition

The prevalence of *Mycobacterium tuberculosis* complex was defined as the proportion of individuals with an evaluable sample (i.e. excluding those where the sample leaked, was missing or all samples were contaminated) from which *Mycobacterium tuberculosis* complex was grown from the original sputum sample. We will hereafter refer to this as prevalent *M.tuberculosis*. We did not exclude those individuals on tuberculosis treatment and so if *M.tuberculosis* was grown, regardless of treatment status, the individual was classified as having prevalent *M.tuberculosis.* However if an individual on tuberculosis treatment was not culture positive for *M.tuberculosis* then they were not considered to have prevalent *M.tuberculosis*.

#### Clinical Definitions

Tuberculosis disease was defined according to the algorithm (Fig. 1). Any individual who had *M.tuberculosis* isolated from their original culture and had at least one positive smear (≥3 AFB seen) or another culture was categorised as having bacteriologically confirmed TB disease. An individual with *M.tuberculosis* isolated from the original culture and who was started on TB treatment due to clinical symptoms or X-ray findings, but did not have any additional microbiological proof of TB, was categorised as having bacteriologically unconfirmed TB disease. Any individual with *M.tuberculosis* isolated from the original culture but with subsequently negative smears and cultures, and who did not have signs and symptoms of TB clinically or on chest x-ray was categorised as having sub-clinical TB [22].

#### TB suspect

Any individual coughing for 3 weeks or more, or with haemoptysis was regarded as a TB suspect according to international standard definitions at the time of the study.

### Data analysis

#### Stata Version 9.0 was used for statistical analysis

Primary analysis of TB and HIV point prevalence and 95% confidence intervals (95%CI) was done using appropriate generalized linear models with random effects to control parameter estimates and standard errors for the effect of clustering at the standard enumeration area. Additionally these models were applied to perform univariate and multivariate analyses. Firstly, possible associated risk factors were explored using univariate regression. Significantly associated risk factors identified at this stage were then introduced into a respective multivariate model. Variables were retained in the final model if associated likelihood-ratio test showed a *P* value<0.2.

The impact of HIV on tuberculosis at community level, assuming causality was estimated by calculating the population attributable fraction.

Sensitivities and specificities of the different screening algorithms, using culture positive *M.tuberculosis* as the gold standard, were calculated with binomial exact confidence intervals.

## Results

**Figure 2:**
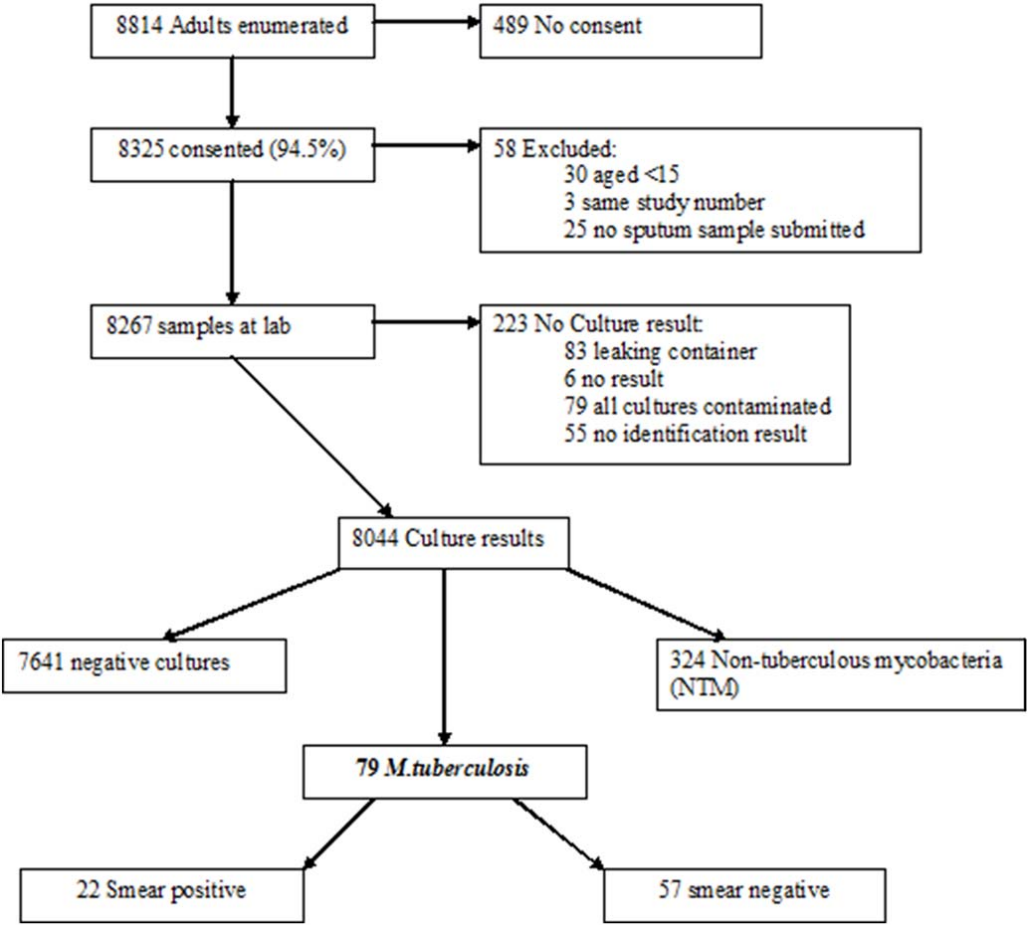
Flowchart of Individuals within the Study.

4048 households with 8814 adults were enumerated from 24 census enumeration areas in the two study sites. 8325 (94.5%) adults consented to be part of the study, 8044 (91.3% of all enumerated) had a full culture result available with 281(3.2% of those enumerated) being excluded for reasons shown in Fig. 2.

Of 8044 evaluable results, 79 had a culture positive for *M.tuberculosis* complex giving a crude prevalence of 980/100,000 and an adjusted prevalence of 870/100,000 (95% CI 570–1160). Of the 79 cases of culture positive *M.tuberculosis 22* (28%) were also smear positive. Of these 8 (36%) were classified as scanty, and 14 (64%) as 1–3+ positive. Since we did not examine the smears of individuals with negative culture results this is a minimum estimate of the smear positive prevalence.

Spoligotyping followed by RFLP analysis of positive cultures found identical fingerprints in 1 pair of samples giving a potential cross-contamination rate of less than 2%.

### Associations with prevalent *M.tuberculosis*

The two communities had significantly different prevalence rates with the more rural community having an adjusted prevalence of 650/100,000 (95% CI 360–940) and the more urban community having a prevalence of 1200/100,000 (95% CI 750–1640) (Table 1).

**Table 1:** Associations with Prevalent *M.tuberculosis*

Characteristic	N	TB	Adj-Prev^1^	95% CI	Univariate			Multivariate		
					OR	95% CI	*p*	OR	95% CI	*p*
Total	8044	79	0.87	0.57–1.16						
Community							0.037			0.0209
*Rural*	4055	28	0.65	0.36–0.94	1.00			1.00		
*Urban*	3989	51	1.20	0.75–1.64	1.86	1.07–3.24		2.06	1.16–3.64	
*Missing*	0									
Age							0.000			0.0018
* 15–24*	3244	15	0.41	0.18–0.65	1.00			1.00		
* 25–34*	1937	22	1.01	0.51–1.5	2.45	1.26–4.74		1.82	0.91–3.64	
* 35–44*	1154	27	2.15	1.20–3.1	5.29	2.79–10.02		3.31	1.67–6.56	
* 45–54*	685	8	1.02	0.27–1.78	2.49	1.05–5.91		1.54	0.62–3.81	
* 55–64*	357	1	0.25	0.00–0.74	0.60	0.08–4.56		0.31	0.04–2.41	
* >64*	327	3	0.83	0.00–1.8	2.01	0.58–7.03		0.98	0.27–3.57	
* Missing*	340	3								
Sex							0.463			
* Male*	3709	39	0.94	0.56–1.32	1.00					
* Female*	4334	40	0.80	0.47–1.13	0.85	0.54–1.32				
* Missing*	1	0								
HIV-result							<0.001			0.0007
* Negative*	5666	36	0.55	0.32–0.79	1.00			1.00		
* Positive*	2297	43	1.65	1.00–2.31	3.03	1.93–4.74		2.30	1.42–3.74	
* Missing*	81	0								
Previous TB							0.008			
* No*	7701	70	0.81	0.53–1.08	1.00					
* Yes*	333	9	2.36	0.71–4.01	2.98	1.47–6.03				
* Missing*	10	0								
Currently Coughing							<0.001			<0.001
* No*	6124	26	0.37	0.20–0.55	1.00			1.00		
* Yes between 1–21 days*	1274	17	1.23	0.56–1.89	3.31	1.78–6.15		3.07	1.62–5.81	
* Yes>21 days*	493	34	6.34	3.71–8.97	18.04	10.60–30.71		12.72	7.05–22.94	
* Missing duration*	153	2								
Shortness breath							<0.001			
* No*	6650	44	0.56	0.32–0.79	1.00					
* Yes*	1384	35	2.28	1.31–3.25	4.17	2.64–6.57				
* Missing*	10	0								
Fever							<0.001			0.0061
* No*	6066	37	0.50	0.27–0.72	1.00			1.00		
* Yes*	1962	42	1.92	1.13–2.71	3.91	2.48–6.16		2.04	1.23–3.39	
* Missing*	16	0								
Night sweats							<0.001			
* No*	6001	40	0.57	0.33–0.81	1.00					
* Yes*	2018	39	1.73	1.02–2.43	3.07	1.96–4.81				
* Missing*	25	0								
Weight loss							<0.001			
* No*	4878	34	0.59	0.33–0.85	1.00					
* Yes*	2868	44	1.36	0.83–1.9	2.33	1.48–3.67				
* Missing*	298	1								
Chest pain							<0.001			
* No*	5886	38	0.56	0.33–0.80	1.00					
*Yes*	2125	40	1.68	1.00–2.35	3.01	1.92–4.72				
* Missing*	33	1								
Car							0.158			
* No*	7697	78	0.90	0.59–1.2	1.00					
* Yes*	330	1	0.28	0.00–0.84	0.31	0.04–2.26				
* Missing*	17	0								
Bicycle							0.061			
* No*	5639	64	1.00	0.65–1.36	1.00					
* Yes*	2388	15	0.60	0.27–0.92	0.59	0.33–1.05				
* Missing*	17	0								
Television							0.927			
* No*	5755	57	0.87	0.55–1.20	1.00					
* Yes*	2272	22	0.85	0.43–1.28	0.98	0.58–1.63				
* Missing*	17	0								
Radio							0.399			
* No*	2693	31	0.99	0.55–1.43	1.00					
* Yes*	5339	48	0.81	0.51–1.11	0.82	0.51–1.30				
* Missing*	12	0								
Fridge							0.319			
* No*	7031	72	0.91	0.59–1.22	1.00					
* Yes*	984	7	0.61	0.12–1.11	0.67	0.30–1.52				
* Missing*	29	0								
Number of meals							0.252			
* 0*	96	2	1.28	0.36–2.19	1.00					
* 1*	1050	13	1.08	0.55–1.6	0.85^2^	0.63–1.13				
* 2*	3152	30	0.91	0.60–1.22						
* 3 or more*	3687	33	0.76	0.45–1.07						
* Missing*	59	1								

1 Adjusted for clustering.

2 OR for trend.

The prevalence of *M.tuberculosis* was highest in the 35–44 years age group (adj OR comparing 35–44 years with 15–24 years 3.31, 95% CI 1.67–6.56) but there was no difference seen between the prevalence in men and women. The prevalence of *M.tuberculosis* was significantly higher in HIV positive individuals than those who were HIV negative (Adj OR 2.30, 95% CI 1.42–3.74).

On univariate analysis there was a significant increased risk of prevalent M.tuberculosis amongst those who had previously had TB (Univariate OR 2.98, 95% CI 1.47–6.03). However this association had significant interaction with HIV status; HIV negative individuals having an increased risk of prevalent *M.tuberculosis* if they had had previous TB compared to the group who had not had previous TB (OR 8.37, 95%CI 3.60–19.48), whereas it appeared protective for HIV-positive individuals (OR 0.58, 95%CI 0.05–1.40), and thus it was not included in the final multivariate model.

We used a simple asset register and the number of meals eaten in a day to give a crude indication of socio-economic status. For any one of these assets there was a trend towards ownership of assets, and therefore higher socio-economic status, decreasing the risk of prevalent *M.tuberculosis* but none was significant.

Of the 79 cases of prevalent *M.tuberculosis* only 51 (65%, 2 missing data) admitted to having any cough and of these 34 (67%) had been coughing for more than 3 weeks (definition for being a TB suspect at the time of this study and needing investigation for tuberculosis). Having a cough of short duration (<21 days) was a risk factor for having prevalent *M.tuberculosis* (adj OR 3.07, 95%CI 1.62–5.81) but coughing for more than 3 weeks provided the strongest association with prevalent *M.tuberculosis* (adj OR 12.72, 95% CI 7.05–22.94). All other symptoms were associated with prevalent *M.tuberculosis* on univariate analysis, but on multivariate analysis only fever remained significant in the final model (Adj OR 2.04, 95%CI 1.23–3.39).

### Clinical Tuberculosis

Following the algorithm shown in Fig. 1, we determined the TB disease status of all individuals from whom *M.tuberculosis* was isolated. Of the 79 individuals with prevalent *M.tuberculosis*, 42 (53%) were bacteriologically confirmed to have TB disease with either subsequent smears or cultures positive for TB, 2 (2.5%) had clinical signs of TB or chest x-ray changes suggestive of TB (and they were started on TB treatment) but the diagnosis was not subsequently confirmed bacteriologically, and 15 (19%) were categorised as having sub-clinical TB. Of these sub-clinical TB cases 4 (27%) had no symptoms at the time of sputum submission, 3 (75%) were HIV positive. By the time of follow up the 3 HIV positive individuals had developed minor symptoms but 2 further sputum smears and cultures were negative for TB. The HIV-negative individual remained asymptomatic and further smears and cultures were negative. This case was one of the pair with the same fingerprint and may represent laboratory error or contamination.

The remaining 11 (73%) had symptoms at the time of the original sputum collection but these had resolved or changed by the time of clinical follow up. Of this group 9 (82%) were HIV-positive and symptoms were often non-specific e.g. fever and weight loss. Chest x-rays were not considered to show tuberculosis and subsequent smears and cultures were negative.

Unfortunately 20 individuals (25%) could not be categorised as they did not complete the clinical follow up because they had died (4, 20%), moved (5, 25%) refused follow up (3, 15%) could not be found (4, 20%) or did not complete the follow-up evaluation (4, 20%).

### HIV

Overall HIV results are available for 7963 of the 8044 individuals included in this analysis (99%). Adjusted HIV prevalence was 28.61% (95% CI 26.04–31.19) in the two sites studied, with no statistical differences being seen between the urban and rural sites (Table 2). HIV prevalence varied with age and sex as has been previously demonstrated in Sub-Saharan Africa being significantly higher in women than in men (Adj OR 1.32 95% CI 1.19–1.47). Having been previously been diagnosed with TB was significantly associated with being HIV positive. The number of symptoms and specific symptoms associated with TB were found more often in HIV positive individuals. The study was not designed to look for specific risk factors for HIV infection, however there was a trend towards owning more assets and having more meals per day, higher socio-economic status, being protective for HIV (e.g. Adj OR for bicycle ownership 0.79, 95% CI 0.7–0.89).

**Table 2:** Associations with HIV

Characteristic	N	HIV	Adj. prev^1^	95% CI	Univariate			Multivariate		
					OR	95% CI	*p*	OR	95% CI	*p*
Total TB-prevalence survey	8044									
HIV-test done	7963	2297	28.61	26.04–31.19						
Community							0.792			
* Rural*	4016	1198	28.94	25.19–32.68	1.00					
* Urban*	3947	1099	28.23	24.34–32.11	0.97	0.74–1.25				
Age							<0.001			<0.001
* 15–24*	3220	630	19.67	17.10–22.25	1.00			1.00		
* 25–34*	1919	705	38.51	34.41–42.61	2.56	2.24–2.92		2.38	2.09–2.73	
* 35–44*	1138	418	38.75	34.19–43.32	2.58	2.22–3.01		2.37	2.02–2.77	
* 45–54*	674	225	35.62	30.63–40.61	2.26	1.88–2.72		2.05	1.69–2.48	
* 55–64*	353	123	36.26	30.15–42.36	2.32	1.83–2.95		2.11	1.65–2.70	
* >64*	324	92	30.24	24.24–36.23	1.77	1.36–2.30		1.55	1.18–2.03	
* Missing*	335	104								
Sex							<0.001			<0.001
* Male*	3670	968	25.97	23.33–28.60	1.00			1.00		
* Female*	4293	1329	30.88	28.05–33.71	1.27	1.15–1.41		1.32	1.19–1.47	
TB-culture							<0.001			0.003
* No Growth*	7562	2153	28.18	25.71–30.63	1.00			1.00		
* MOTT*	322	101	30.60	24.92–36.27	1.12	0.88–1.44		1.13	0.86–1.48	
* TB*	79	43	54.83	43.49–66.18	3.09	1.97–4.85		2.25	1.39–3.66	
Previous TB										<0.001
* No*	7621	2124	27.60	25.19–30.01	1.00		<0.001	1.00		
* Yes*	333	170	51.78	45.70–57.86	2.82	2.25–3.52		2.31	1.83–2.93	
* Missing*	9	3								
TB-Suspect							<0.001			<0.001
* No*	7385	2067	27.86	25.57–30.15	1.00			1.00		
* Yes*	578	230	40.53	35.71–45.35	1.76	1.48–2.11		1.44	1.19–1.76	
Number of symptoms							<0.001			
* 0*	2685	664	24.84	22.67–27.01	1.00					
* 1 ^3^*	2021	561	27.46	25.32–29.60	1.15	1.11–1.18				
* 2*	1330	395	30.24	27.98–32.51						
* 3*	892	271	33.18	30.61–35.74						
* 4*	534	199	36.25	33.22–39.28						
* 5*	305	118	39.44	35.83–43.05						
* 6*	196	89	42.73	38.46–46.99						
Car							0.186			
* No*	7617	2205	28.75	26.16–31.34	1.00					
* Yes*	329	87	25.41	20.21–30.60	0.84	0.65–1.09				
* Missing*	17	5								
Bicycle							<0.001			<0.001
* No*	5577	1685	29.98	27.76–32.20	1.00			1.00		
* Yes*	2369	605	24.56	22.16–26.97	0.76	0.68–0.85		0.79	0.70–0.89	
* Missing*	17	7								
Television							0.011			
* No*	5698	1694	29.66	27.03–32.28	1.00					
* Yes*	2248	598	26.55	23.86–29.25	0.86	0.76–0.97				
* Missing*	17	5								
Radio							0.760			
* No*	2676	775	28.84	25.87–31.80	1.00					
* Yes*	5275	1518	28.47	25.80–31.15	0.98	0.88–1.09				
* Missing*	12	4								
Fridge							0.225			
* No*	6964	2028	28.97	26.33–31.61	1.00					
* Yes*	970	262	26.89	23.33–30.45	0.90	0.76–1.07				
* Missing*	29	7								
Number of meals							<0.001			0.004
* 0*	95	41	36.67	32.20–41.13	1.00			1.00		
* 1 ^3^*	1035	350	33.06	29.87–36.25	0.85^2^	0.80–0.91		0.90^2^	0.84–0.97	
* 2*	3127	897	29.64	27.22–32.06						
* 3 or more*	3648	994	26.44	24.07–28.81						
* Missing*	58	15								

1 adjusted for clustering.

2 OR for trend.

The HIV prevalence among individuals with prevalent *M.tuberculosis* was 55%. Assuming causality, the impact (population attributable fraction, PAF) of HIV on prevalent TB in these communities was 36.0%.

We identified a large number of non-tuberculous mycobacteria (NTM), which will be reported in a separate publication.

### Symptoms

Overall 5319 (66%) of the population questioned declared at least one symptom, and 1920 (23.8%) had a cough. Only 581 (7.2%) met the definition of a suspect who should be further investigated for tuberculosis. Despite the surprisingly high proportion of symptomatic individuals in the community, 8 of the 79 (10%) individuals with *M.tuberculosis* isolated from their sputum denied having any symptoms at all, and only 34 (43%) would be classified as a TB suspect.

Taking any symptom into consideration, the more symptoms an individual had the more likely they were to have *M.tuberculosis* isolated from their sputum (OR for trend 1.71, 95% CI 1.52–1.93) and the more likely they were to be HIV positive (OR for trend 1.15, 95% CI 1.11–1.18). Apart from cough the symptoms most associated with a diagnosis of prevalent *M.tuberculosis* were different in HIV positive compared to HIV negative individuals. For HIV negative individuals cough for 1–3 weeks was a risk factor for prevalent *M.tuberculosis* (Adj OR 3.96, 95% CI 1.57–10.00) as was cough for 3 weeks or more (Adj OR 11.18, 95% CI 4.45–28.07) and fever (Adj OR 2.63, 95% CI 1.23–5.63). For HIV positive individuals only cough of 3 weeks or more (Adj OR 11.95, 95% CI 5.38–26.56) and chest pain (Adj OR 2.11, 95%CI 1.23–5.63) were significantly associated with having prevalent *M.tuberculosis*.

If the standard definition of a TB suspect, i.e. cough for 3 weeks or more or haemoptysis, is used in these communities it gives a sensitivity of 43.4% and specificity of 93.1% for the whole population (Table 3). The sensitivity and specificity compared by HIV status shows higher values in HIV positive individuals but these are generally not statistically significant. Modifying the algorithm to include either cough for more than 3 weeks/haemoptysis or any other 2 symptoms improves the sensitivity to 74.7%.

**Table 3:** Sensitivity, specificity and predictive values for different screening algorithms

	Sensitivity	Specificity	PPV	NPV
Any symptom				
All	89.9 (81.0–95.5)	34.1 (33.1–35.2)	1.3 (1.0–1.7)	99.7 (99.4–99.9)
HIV Negative	83.3 (67.2–93.6)	35.8 (34.5–37.1)	0.8 (0.6–1.2)	99.7 (99.4–99.9)
HIV Positive	95.3 (84.2–99.4)	29.4 (27.5–31.3)	2.5 (1.8–3.4)	99.7 (98.9–100)
Any Cough				
All	67.1 (55.6–77.3)	76.6 (75.6–77.5)	2.8 (2.1–3.6)	99.6 (99.4–99.7)
HIV Negative	66.7 (49.0–81.4)	77.8 (76.7–78.9)	1.9 (1.2–2.8)	99.7 (99.5–99.9)
HIV Positive	67.4 (51.4–80.9)	73.1 (71.3–75.0)	4.6 (3.1–6.5)	99.2 (98.6–99.5)
TB Suspect (Cough 3 weeks or more or haemoptysis)				
All	43.0 (31.9–54.7)	93.1 (92.6–93.7)	5.9 (4.1–8.1)	99.4 (99.2–99.6)
HIV Negative	36.1 (20.8–53.8)	94.0 (93.4–94.7)	3.7 (2.0–6.3)	99.6 (99.4–99.7)
HIV Positive	48.8 (33.3–64.5)	90.7 (89.5–91.9)	9.1 (5.7–13.6)	98.9 (98.4–99.3)
TB suspect or any other 2 symptoms				
All	74.7 (63.6–83.8)	58.8 (57.7–59.9)	1.8 (1.3–2.3)	99.6 (99.3–99.7)
HIV Negative	69.4 (51.9–83.7)	61.0 (59.7–62.2)	1.1 (0.7–1.7)	99.7 (99.4–99.8)
HIV Positive	79.1 (64.0–90.0)	53.0 (51.0–55.1)	3.1 (2.2–4.3)	99.3 (98.6–99.7)

## Discussion

This paper presents data from a large community based study, with samples collected from a high proportion of targeted adults. The estimated prevalence of tuberculosis of 870/100,000 adults is high, but it is in line with the WHO estimation of a prevalence of 707/100,000.

Few large-scale TB and HIV prevalence surveys have been conducted in Africa. Previous studies in the pre-HIV era and in lower HIV prevalence settings have estimated a lower prevalence of TB but comparisons must be made with caution as differences in sampling, screening strategies and also the laboratory methods used, both in the smear and culture procedures, make direct comparisons difficult [26]. The only other prevalence surveys where all participants had a TB culture performed show a similar range of prevalent TB [14], [15], [20], [21], higher in the gold mines of South Africa[14] and in a high prevalence township in the Western Cape [21]- although the confidence intervals surrounding this estimate are wide due to the small sample size (Table 4).

**Table 4:** Table of Surveys where all participants were cultured

Study Ref	Year	Country	Population	Number in analysis	Culture method	Culture prevalence /1000^1^	Smear prevalence /1000^1^	HIV+ve culture prev	HIV−ve culture prev
Corbett	2001	S. Africa	Miners	1773	LJ	45/1734	9/1734	17/453	28/1281
						26.5	5.2	37.5	21.9
Corbett	2002	Zimbabwe	Factory workers	4668	LJ/PNB	15/4668	6/4668	5/874	10/3974
						3.2	1.3	5.7	2.5
Den Boon	2002	S. Africa	Community	2608	LJ	26/2608	8/2608	N/a	N/a
						10	3.1		
Wood	2005	S. Africa	Community	762	MGIT/PNB/pcr	12/762	6/762	9/174	3/588
						15.7	7.9	51.7	5.1
Ayles	2005	Zambia	Community	8044	MGIT/LJ/Hain	79/8044	22/8044	43/2855	36/5630
						9.8	2.7	15.1	6.4

1 Crude prevalence per 1000 population surveyed

In this study HIV had a significant effect on the prevalence of tuberculosis. This contrasts to the two studies conducted in occupational settings where HIV was found to influence incidence of disease much more than prevalence [14], [15]. This difference may reflect the better access to medical services afforded to individuals in such occupational settings. Indeed it is possible that HIV is having a double effect on TB control services in our communities; not only are there more TB cases and therefore increased transmission of disease, but also there is an increasing burden on over-stretched health services, making effective control harder to achieve [27]. Our study found many cases of undiagnosed tuberculosis in the community but it is unclear how many would have eventually received treatment in the absence of the survey. In our definition of prevalent tuberculosis we only included those individuals with a culture positive for *M.tuberculosis*, and excluded those individuals who were currently on TB treatment but whose culture was negative, however 5 of our prevalent cases reported that they were already on TB treatment at the time of the survey.

Although tuberculosis was strongly associated with coughing for 3 weeks or more in both HIV negative and HIV positive individuals, the study demonstrates that many individuals have culture positive tuberculosis in the absence of symptoms sufficient to meet the definition of being a TB suspect. This study gives more insight into the effectiveness of symptom screening at community level than previous studies. The definition of a TB suspect, i.e. cough for more than 3 weeks or haemoptysis has a sensitivity of 48.8% in HIV positive individuals, but this can be improved by considering other symptoms. If any symptom is considered the sensitivity becomes 95.3% but such a strategy would result in a very large number of the HIV-positive population requiring laboratory investigation, requiring significant laboratory expansion and strengthening. A more practical approach may be to use an algorithm that includes traditional TB suspects or anyone with at least 2 other symptoms, this increases the sensitivity to 79.1% while reducing the burden on the laboratory. If we consider 1000 HIV positive individuals, our data indicate that 476 would be classified as TB suspects under this scenario and would therefore require additional laboratory tests to identify a possible 15 cases of culture positive *M.tuberculosis* (i.e. 32 individuals would need to be investigated for every positive case). Since conducting this study the definition of a TB suspect has been changed to include all individuals coughing for 2 weeks or more. We did not include this question in our questionnaire and so are unable to comment on how this would have performed in this setting.

The findings of this study have significant implications for TB-HIV activities as are recently highlighted by WHO HIV departments “3I's” initiative [28] (Intensified case finding, Isoniazid preventive therapy and Infection Control) and TB department (Interim Policy for collaborative TB-HIV activities) [4]. This study demonstrates a very high burden of both TB and HIV in these Zambian communities. Intensified case finding for both TB and HIV could have a significant impact on both epidemics, especially if treatment for both conditions was universally available [29]. It may be that community level approaches could reduce transmission of TB and HIV and despite logistical challenges would be worthwhile in the long term. The widespread use of ART may have an impact on the prevalence of tuberculosis. Mathematical models show that as more HIV-positive individuals live for longer with some degree of immunosuppression, the burden of tuberculosis may increase despite the fact that the incidence of tuberculosis in these individuals will fall [30]. Our study was conducted at a time when ART use was low at community level and so does not give any insight into this but in future ART usage should be recorded in prevalence surveys.

Isoniazid preventive therapy has been demonstrated to reduce the risk of HIV-positive individuals developing tuberculosis but active tuberculosis must be excluded before it is used [31]. What screening is necessary to exclude tuberculosis has been controversial, however the negative predictive value of a screen is the most important statistic to give an indication of the proportion of cases that would be missed by any particular screen. Our data show that at the community level the negative predictive value of any of the screens is high and so the number of cases of active tuberculosis that would be missed is low. If a modified screen consisting of either the traditional TB suspect definition, or any other 2 symptoms is used, the negative predictive value in HIV-positive individuals is 99.3%. In a hypothetical scenario where 1000 HIV-positive individuals from a similar community to the ones described in this paper are screened, 524 individuals would be declared free of symptoms indicating active TB and could potentially be started on isoniazid preventive therapy. Of these 524, 4 would actually have a culture positive for TB. All four would have negative sputum smears and therefore probably represent paucibacillary disease which may actually respond to mono-therapy with isoniazid although they may also develop into isoniazid resistant cases. It is not known how many such cases it is acceptable to miss and the likelihood of generating isoniazid resistance in these cases so further research is needed of large-scale implementation. What is known is that over the next two years, the isoniazid preventive therapy would prevent about 15 new cases of tuberculosis [32].

Infection control is the third of the 3I's. Whilst most of the cases of prevalent tuberculosis were not smear positive, and therefore not the most infectious, we have to assume that all of these cases were capable of transmitting tuberculosis within the community and especially to other vulnerable individuals, including those with HIV [33]. Infection control in congregate settings is critical, especially those where HIV positive individuals spend large amounts of time such as health centres. It may be that screening of all individuals with any symptom in these settings would be warranted and that symptomatic individuals need to be separated from others.

Since this survey was done the WHO definition for tuberculosis disease has been changed to include all individuals with a single positive smear or culture, rather than the requirement for at least 2 positive results. This raises interesting questions as 14 of the individuals that we identified did not have further confirmation of disease and were not started on TB treatment. There are several hypotheses to account for what we have termed sub-clinical TB disease. Some of these cases might represent laboratory contamination or sample mislabelling (as appears to be the case for 1 individual). However our quality control data show that laboratory contamination was rare and that this could not account for the other cases identified. All samples were labelled with barcodes and scanners were used to minimise the chances of transcription errors, but again this would not account for all the cases identified. It could be that some of these individuals are transiently excreting *M.tuberculosis* but then either self-cure or that we are in fact picking up primary infections that are being contained and converted into a latent infection. These hypotheses are difficult to prove although some of these individuals were symptomatic at first encounter and when they were re-visited their symptoms had resolved and cultures were negative. It may be that with expanded use of the newer, more sensitive liquid culture techniques that we used in this survey this phenomenon will become more apparent and a recent study found similar cases in Tanzania [22].

### Study Limitations

This was a large and challenging study and therefore the procedures and questionnaires had to be kept as simple as possible. Some areas where more detail would have been desirable include socioeconomic status and more detailed questions about duration of symptoms. A follow up nested case-control study, specifically looking at the relationship between socioeconomic status and prevalent TB, will be reported elsewhere.

The laboratory work involved in this study was extremely time consuming with relatively few positive results to be found. It would have been desirable to examine sputum smears on all individuals but due to a lack of human resources necessary to screen over 8000 slides where more that 99.5% are expected to be negative, only the slides from the culture positive cases were examined. This means that we have a minimum estimate of the prevalence of tuberculosis as some cases may not have grown in culture due to overly harsh decontamination leading to mycobacterial killing. Our definition of both smear positivity (agreement by 2 teams of 2 readers) and clinical TB were strict, again minimising the estimate of prevalence.

### Conclusions

This study demonstrates that prevalence of tuberculosis is high in Zambia and that 36% of all prevalent tuberculosis in these communities is attributable to HIV. HIV appears to have overshadowed previously described risk factors for prevalent tuberculosis such as male sex, urban environment and previous TB such that it is now the major driving force behind prevalent TB.

The widely used screening algorithms are not sufficient to find all cases of tuberculosis, but it may not be feasible to use other more sensitive screens until better diagnostic tests become available. Despite this, community case finding for TB and HIV would detect a large proportion of currently undetected cases more quickly and therefore limit transmission. Symptom screening can rule tuberculosis out with a high negative predictive value and could therefore be useful in exclusion of tuberculosis prior to provision of isoniazid preventive therapy.

If Zambia, and the rest of Africa, is to meet the Millennium Development Goals with respect to tuberculosis and HIV we need to do much more to find cases and treat them, therefore interrupting transmission at community level.
